# Efficacy of sertraline against *Trypanosoma cruzi*: an in vitro and in silico study

**DOI:** 10.1186/s40409-018-0165-8

**Published:** 2018-10-30

**Authors:** Daiane Dias Ferreira, Juliana Tonini Mesquita, Thais Alves da Costa Silva, Maiara Maria Romanelli, Denise da Gama Jaen Batista, Cristiane França da Silva, Aline Nefertiti Silva da Gama, Bruno Junior Neves, Cleber Camilo Melo-Filho, Maria de Nazare Correia Soeiro, Carolina Horta Andrade, Andre Gustavo Tempone

**Affiliations:** 1 Instituto Adolfo Lutz, Centre for Parasitology and Mycology, Avenida Dr. Arnaldo 351, 8° andar, sala 9, CEP, São Paulo, SP 01246-000 Brazil; 2 Fundação Oswaldo Cruz, Laboratório de Biologia Celular do Instituto Oswaldo Cruz, Av. Brasil, 4365 Manguinhos, CEP, Rio de Janeiro, RJ 21040-360 Brazil; 3 Faculdade de Farmácia, Universidade Federal de Goiás, Rua 240 Setor Leste Universitário, Goiânia, GO 74605170 Brazil

**Keywords:** *Trypanosoma cruzi*, Drug, Treatment, Sertraline, Drug repurposing, Drug repositioning

## Abstract

**Background:**

Drug repurposing has been an interesting and cost-effective approach, especially for neglected diseases, such as Chagas disease.

**Methods:**

In this work, we studied the activity of the antidepressant drug sertraline against *Trypanosoma cruzi* trypomastigotes and intracellular amastigotes of the Y and Tulahuen strains, and investigated its action mode using cell biology and in silico approaches.

**Results:**

Sertraline demonstrated in vitro efficacy against intracellular amastigotes of both *T. cruzi* strains inside different host cells, including cardiomyocytes, with IC_50_ values between 1 to 10 μM, and activity against bloodstream trypomastigotes, with IC_50_ of 14 μM. Considering the mammalian cytotoxicity, the drug resulted in a selectivity index of 17.8. Sertraline induced a change in the mitochondrial integrity of *T. cruzi*, resulting in a decrease in ATP levels, but not affecting reactive oxygen levels or plasma membrane permeability. In silico approaches using chemogenomic target fishing, homology modeling and molecular docking suggested the enzyme isocitrate dehydrogenase 2 of *T. cruzi* (*Tc*IDH2) as a potential target for sertraline.

**Conclusions:**

The present study demonstrated that sertraline had a lethal effect on different forms and strains of *T. cruzi*, by affecting the bioenergetic metabolism of the parasite. These findings provide a starting point for future experimental assays and may contribute to the development of new compounds.

**Electronic supplementary material:**

The online version of this article (10.1186/s40409-018-0165-8) contains supplementary material, which is available to authorized users.

## Background

Chagas disease is a neglected tropical protozoan disease that affects 8 million people, mainly in South and Central America [1]; however, cases have also been reported in North America, Europe and some other Western countries [2]. The current treatment of Chagas disease is an alarming issue. A recent clinical study re-evaluated the efficacy of benznidazole (BZ), the only available treatment in Brazil; despite a reduction in parasitemia, the study revealed that BZ induced no significant improvements in cardiac clinical outcomes during chronic-phase Chagas disease [3]. According to the non-profit organization Drugs for Neglected Diseases initiative (DNDi), in the next 5 years, 200,000 people living with Chagas disease will die from heart disease and related complications. DNDi also highlighted the urgent need to find better treatments [4]. Additionally, the drug safety of the available treatment is inadequate; BZ is implicated in serious systemic side effects, including anorexia, vomiting, peripheral polyneuropathy, depression of bone marrow, and allergic dermopathy. As a consequence of these adverse reactions, treatment has to be discontinued [5].

The discovery of new therapeutic agents is expensive and may take many years. Several strategies have been implemented in order to reduce the costs and time of the drug discovery process. Drug repositioning has emerged as a promising strategy for drug discovery for Neglected Tropical Diseases (NTDs). Also referred to as drug repurposing, this strategy consists of finding novel indications for approved drugs [6]. Sertraline (SERT), a serotonin reuptake inhibitor, is widely used in the treatment of patients with depression and severe anxiety disorders [7], and has exhibited antifungal [8], antimicrobial [9], and anticancer activities [10]. The drug has shown promising activity against the protozoan *Leishmania donovani*; it reduced the parasite burden of animals by 72% at doses of 10 mg/kg for 30 days [11].

In the present work, we evaluated the activity of SERT against *T. cruzi* trypomastigotes and intracellular amastigotes of the Y and Tulahuen strains and investigated its mode of action using cell biology and in silico chemogenomic approaches.

## Methods

Additional file 1 shows a Flowchart of the global methodology.

### Animals

BALB/c mice were supplied by the animal breeding facility at the Adolfo Lutz Institute of São Paulo whereas Swiss mice were obtained from the Fundação Oswaldo Cruz (FIOCRUZ) Rio de Janeiro. The mice were maintained in sterilized cages under a controlled environment and received water and food ad libitum. Animal procedures were performed with the approval of the Research Ethics Commission, in agreement with the Guidelines for the Care and Use of Laboratory Animals from the National Academy of Sciences. All procedures carried out at Institute Adolfo Lutz were approved by the Committee for Ethics in the Use of Animals (CEUA 04/2016). All procedures performed at FIOCRUZ were in accordance with the guidelines established by the FIOCRUZ Committee for Ethics in the Use of Animals (CEUA LW16/14).

### Drugs and chemicals

Resazurin, Roswell Park Memorial Institute medium (RPMI 1640) without phenol red, and Hanks’ Balanced Salt Solution (HBSS), were purchased from Sigma-Aldrich. Sytox Green® and H2CDFDA (2 ‘, 7′-dichlorodihydrofluorescein diacetate) were purchased from Molecular Probes ® (Invitrogen ™). Fetal Bovine Serum (FBS) was obtained from Gibco and gentamicin sulfate from Hipolabor Pharmaceuticals. Benznidazole (BZ) and sertraline (SERT) were purchased from Sigma-Aldrich. All other reagents not mentioned were purchased from Sigma-Aldrich.

### Parasites and mammalian host cells

#### *T. cruzi* (Y strain - culture trypomastigotes)

Trypomastigotes were maintained in LLC-MK2 cells with RPMI-1640 medium supplemented with 2% fetal bovine serum (FBS) at 37 °C and 5% CO_2_ in a humidified incubator.

#### *T. cruzi* (Y strain - bloodstream trypomastigotes - BT)

Trypomastigotes were obtained from the blood samples of infected albino Swiss mice at the peak of parasitemia. The purified parasites were resuspended in Dulbecco’s Modified Eagle Medium (DMEM) supplemented with 10% FBS as reported previously [12].

#### Macrophages

Macrophages used in the intracellular amastigote assays were collected from the peritoneal cavity of BALB/c mice by washing with RPMI-1640 medium supplemented with 10% FBS and maintained at 37 °C in an atmosphere of 5% CO_2_ in a humidified incubator.

#### Cardiac cell cultures (CC)

Cardiac cells were used in the cytotoxicity and intracellular amastigote assays. Primary cultures of embryonic cardiac cells were obtained from Swiss mice as previously reported [12]. Briefly, after purification, the CC were seeded at a density of (0.2 × 10^6^ cell/well) into 24-well microplates containing gelatin-coated cover slips as previously described. The cardiac cultures were then sustained at 37 °C in DMEM supplemented with 10% horse serum, 5% fetal bovine serum, 2.5 mM CaCl_2_, 1 mM L-glutamine and 2% chicken embryo extract.

#### NCTC cells-clone L929

NCTC cells were maintained in medium M-199 supplemented with 10% FBS and were maintained at 37 °C under 5% CO_2_ in a humidified incubator.

### Determination of the Anti-*T. cruzi* activity

#### Culture trypomastigotes

To determine the 50% inhibitory concentration (IC_50_) against *T. cruzi*, trypomastigotes were counted in a Neubauer hemocytometer and seeded at (1 × 10^6^ cells/well) in 96-well microplates. The drug was dissolved in dimethyl sulfoxide (DMSO), diluted with RPMI-1640 medium at different concentrations for 24 h at 37 °C and placed in a 5% CO_2_ humidified incubator. The parasite viability was determined using the colorimetric resazurin assay [13]. Benznidazole was used as the standard drug. The optical density was read at 570 nm (FilterMax F5 Multi-Mode Microplate Reader, Molecular Devices). DMSO was used at a maximal concentration of 0.5% in all assays and was incubated with cells as an internal control.

#### Bloodstream trypomastigotes (BT)

The trypomastigotes (5 × 10^6^/mL) were incubated for 24 h at 37 °C in RPMI medium in the presence or absence of serial dilutions of the drug (0 to 50 μM). After 24 h of incubation, the parasite death rate was determined by light microscopy through direct quantification of the number of living parasites using a Neubauer chamber, and the IC_50_ was then calculated.

### Determination of cytotoxicity

#### Cytotoxicity against mammalian cells

NCTC cells 929 (6 × 10^4^ cells/well) in 96-well microplates were incubated with the drug for 48 h at 37 °C in a 5% CO_2_ incubator. The selectivity index (SI) was determined using the following formula: CC_50_ against mammalian cells/ IC_50_ against parasites. The cell viability was determined using the colorimetric resazurin assay [13]. The obtained data represent the mean of two independent experiments performed in duplicate.

#### Cytotoxicity against CC

Cardiac cells were incubated at 37 °C for different periods of time (24–48 h) with increasing concentrations of the drug and were diluted in DMEM (without phenol red). The mammalian cell morphology and spontaneous contractibility were evaluated by light microscopy, whereas the cellular viability was determined by the colorimetric resazurin assay. After incubation for 24 h, the absorbance was determined at 570 nm [14]. The obtained data represent the mean of two independent experiments performed in duplicate.

### Intracellular amastigotes of *T. cruzi* in peritoneal macrophages

After the mammalian cytotoxicity studies, the effect of sertraline was investigated against intracellular amastigotes. Peritoneal macrophages (1 × 10^5^ cells/well) were dispensed in 16-well chamber slides (NUNC, Thermo, USA) and maintained for 24 h in the same medium at 37 °C in a 5% CO_2_ humidified incubator for attachment. Non-adherent cells were removed by two-step washings with medium. After 24 h, these cells were infected with (1 × 10^6^ culture trypomastigote) forms for 4 h (parasite-to-macrophage ratio 10:1). Subsequently, infected cells were incubated with the drug for 48 h. Finally, the slides were fixed with methanol, stained with Giemsa, and observed via light microscopy. The parasite load was defined by counting 400 macrophages/well by evaluating the number of infected macrophages. Benznidazole was used as the standard drug. The obtained data represent the mean of two independent experiments performed in duplicate.

### Intracellular amastigotes of cardiac cell cultures

For analysis of the effect against intracellular amastigotes from the Y strain, after 24 h of parasite-host cell interaction, the infected cardiac cell cultures were washed to remove free parasites and were then incubated for another 48 h with increasing concentrations of the drug. Cardiac cell cultures were maintained at 37 °C in an atmosphere of 5% CO_2_ and air, and the medium was replaced every 24 h. Then, untreated and treated infected cardiac cell cultures were fixed and stained with Giemsa solution, and the mean number of infected host cells and mean number of parasites per infected cell were scored. Only characteristic *T. cruzi* nuclei and kinetoplasts were counted as living parasites since irregular structures may indicate parasites undergoing death. The compound activity was estimated by calculating the infection index (II - percentage of infected cells times the average number of intracellular amastigotes per infected host cell) [15]. The obtained data represent the mean of two independent experiments performed in duplicate.

### Intracellular amastigotes inside L929 cell lines

The effect against intracellular forms was also investigated in L929 cell lineages infected with tissue culture-derived trypomastigotes (Tulahuen strain expressing the *Escherichia coli* β-galactosidase gene), employing a parasite-to-host-cell ratio of 10:1. After incubation with the drug for 96 h, the viability of parasites was determined colorimetrically as previously reported [14]. The obtained data represent the mean of two independent experiments performed in duplicate.

### Mode of action studies of SERT in *T. cruzi*

#### Spectrofluorimetric detection of the permeability of the cell membrane

Culture trypomastigotes were washed with PBS (phosphate buffered saline), deposited on a microplate (2 × 10^6^ cells/well) and incubated with SYTOX Green® (1 μM) for 15 min at 24 °C [16]. Sertraline was added at the IC_50_ value (2 μM), and the fluorescence was measured after 20, 40 and 60 min. The maximum permeability was observed with 0.1% Triton X-100 (positive control). The fluorescence intensity was determined using a plate spectrofluorimeter (FilterMax F5 Multi-Mode MicroplateReader-Molecular Devices) with excitation and emission wavelengths of 485 and 520 nm, respectively. Untreated trypomastigotes and 0.5% (*v*/v) DMSO-treated parasites were used in all assays as negative controls. The obtained data represent the mean of two independent experiments performed in triplicate.

#### Effect of sertraline on the mitochondrial integrity

Culture trypomastigotes were washed with PBS, deposited on a microplate (2 × 10^6^ cells/well) and incubated with sertraline at the IC_50_ value (2 μM) for 60 min at 37 °C. MitoTracker Red CM-H_2_XROS (500 nM) was added and the incubation continued for 40 min in the dark. Parasites were washed twice with HBSS (Hanks’ Balanced Salt Solution), and the fluorescence was measured using a plate spectrofluorimeter (FilterMax F5 Multi-Mode Microplate Reader-Molecular Devices) with excitation and emission wavelengths of 540 and 595 nm, respectively [17]. Carbonyl cyanide 4-(trifluoromethoxy)phenylhydrazone (FCCP; 10 μM) was used as a positive control [18]. The obtained data represent the mean of two independent experiments performed in triplicate.

#### Analysis of reactive oxygen species (ROS)

Culture trypomastigotes (2 × 10^6^ cells/well) were washed in HBSS (Hanks’ Balanced Salt Solution) and incubated with sertraline at the IC_50_ value (2 μM) for 60 min at 37 °C. H_2_DCF-DA (5 μM) was added, and the cells were incubated for 15 min. The fluorescence intensity was detected using a plate spectrofluorimeter (FilterMax F5 Multi-ModeMicroplate Reader-Molecular Devices) at 485 and 520 nm for excitation and emission, respectively [19]. The obtained data represent the mean of two independent experiments performed in triplicate.

#### Measurement of cellular ATP content

The intracellular adenosine triphosphate (ATP) content was measured by a luciferin–luciferase bioluminescence assay using a specific kit (Life Technologies, USA) according to the manufacturer’s instructions. ATP concentrations were calculated using the ATP standard curve kit. Culture trypomastigotes were washed twice with PBS, seeded at (2 × 10^6^ cells/well) and incubated at the IC_50_ value (2 μM) of sertraline in the presence or absence (control) of 0.1% Triton X-100 for 1 h. The ATP level was rapidly measured by a coupled luciferin-luciferase reaction [20]. The obtained data represent the mean of two independent experiments performed in triplicate.

### In silico studies

#### Prediction of sertraline targets using publicly available databases

We carried out a literature search using PubMed, PubChem Bioassay, ChEMBL and BindingDB in order to identify all possible SERT targets for all organisms. Our definition of a “sertraline target” embraces in vitro assays of SERT against any enzyme, receptor or channel with inhibition ≤40 μM. Then, individual information for each SERT target (primary amino acid sequence in FASTA format, target name and organism) was obtained from the UNIPROT database and was subsequently allocated into a single Excel file (Additional file 2).

#### Pairwise protein alignment

Superimposed structures allow a comparison of functionally relevant features, conserved residues necessary for catalysis, and residues critical for ligand binding. Therefore, SERT targets were aligned with all *T. cruzi* proteins using pairwise BLAST. We considered the *T. cruzi* target to be druggable if it has ≥80% overlap of the corresponding SERT target and an expected value (E-value) ≤ 10^− 20^. The E-value represents the number of hits with an alignment score “Z” equal to or greater than the “Z” that would be expected by chance when searching a database, which is the expected number of times a homology will occur at random from a given set of trials.

#### Comparison of functional regions

The ConSurf server [21] is a bioinformatics tool for estimating the evolutionary conservation of amino-acid positions in a protein based on the phylogenetic relations between homologous sequences. We used ConSurf for an additional characterization of the functional regions (active site conservation) in *T. cruzi* targets. Therefore, the degree of conservation of the amino acids from the active site was estimated using 150 homologue proteins with similar sequences retrieved from the UNIPROT database and was identified by the PSI–BLAST method (E-value cutoff ≤1^− 10^) [22]. The sequences were clustered, and highly similar (> 95%) sequences were removed using CD-HIT [23]. A multiple sequence alignment (MSA) of the homologous sequences was constructed using MAFFT-L-INS-I [23]. Subsequently, the MSA was employed to construct a phylogenetic tree using the neighbor-joining algorithm [24]. Position-specific conservation scores were analyzed using the empirical Bayesian method. Subsequently, functional regions were visually compared with corresponding SERT targets and were classified as conserved (≥ 70%) or non-conserved (< 70%). The obtained results are described in Additional files 3, 4, 5 and 6.

#### Homology modeling and molecular docking

In the absence of available experimental data, a homology model of *T. cruzi* isocitrate dehydrogenase 2 (*Tc*IDH2) was constructed using homology modeling by comparing the sequence of this target protein with sequences of other proteins (template) for which experimental structures are available. The *Tc*IDH2 sequence was obtained from the TriTrypDB database (Accession: Tc00.1047053506925.319). A BLAST search was carried out with the Protein Data Bank for the identification of a template structure. Three IDH crystal structures were found: human (*Hs*IDH2), *Sus scrofa* and *Mycobacterium tuberculosis* (PDB IDs: 4JA8, 1LWD, 4HCX, respectively). Based on the resolution, the crystal structure of *Hs*IDH2 bound to the allosteric inhibitor AGI-6780 (PDB ID 4JA8) [25], was chosen as a template for homology modeling, which presented 66.5% of sequence identity in relation to *Tc*IDH2. SWISS-MODEL program [26] was used for generation of the homology model of *Tc*IDH2. The quality of the model was assessed using the programs PROCHECK [27], VERIFY 3D [28] and ERRAT.

Molecular docking studies were performed to investigate the intermolecular interactions between SERT and the amino-acid residues of *Tc*IDH2 as well as to predict the binding affinity. The generated homology model of *Tc*IDH2 was imported into Maestro v. 10.0 [29] and was prepared using the Protein Preparation Wizard workflow as follows: hydrogen atoms were added according to Epik v. 2.7 (pH 7.4 ± 1.0) and minimized using the OPLS-2005 force field. Next, the structure of SERT was imported from the ChemSpider database and 300 conformations were generated using OMEGA v. 2.5.1.4 [30]. Subsequently, the conformers had their most favorable ionization state calculated at pH 7.4 using the ‘fixpka’ function, and AM1-BCC charges were added using QUACPAC v.1.6.3.1 [29]. Before the docking studies, two different grids were defined to include the catalytic site and one allosteric site of *Tc*IDH2. The catalytic site grid was built with the dimensions of 26.3 Å × 15.8 Å × 27.9 Å (*x, y* and *z*) and a volume of 11,645 Å^3^. The allosteric site grid had the dimensions of 16.3 Å × 20.3 Å × 19.7 Å and a volume of 6531 Å^3^. Finally, molecular docking of SERT with *Tc*IDH2 was investigated using the software FRED, which is available in OEDocking suite v. 3.0.1 [31] using the high-resolution precision and ChemGauss 4 scoring function.

### Statistical analysis

The obtained data represent the mean of three independent experiments performed in duplicate. The IC_50_ and CC_50_ values were calculated using sigmoid dose-response curves generated by the software GraphPad Prism version 5.0 (GraphPad Software, San Diego, CA, USA). The ANOVA test was performed to evaluate the significance (*p* < 0.05) of data.

## Results

### Antiparasitic activity and mammalian cytotoxicity of sertraline

The anti-trypanosomal activity of SERT in cell culture-derived trypomastigotes (Y strain) was determined colorimetrically by resazurin. After 24 h, 100% of the parasites were eliminated, resulting in an IC_50_ value of 1.8 μM (± 0.8). Using blood-derived trypomastigotes, SERT showed an IC_50_ value of 14.2 μM (± 5.5) (Table 1).

**Table 1:** Antiparasitic activity and mammalian cytotoxicity of sertraline

*T. cruzi* IC_50_ μM (±SD)						CC_50_ μM (±SD)	
Drugs	Trypomastigote (Y)	Trypomastigote BT (Y)	Amastigote L929 (Tulahuen)	Amastigote CC (Y)	Amastigote MØ (Y)	L929	CC
SERT	1.8 ± 0.8	14.2 ± 5.5	10.0 ± 1.7	6.6 ± 1.4	1.4 ± 0.6	11.5 ± 2.4	25.0 ± 13.2
BZ	17.7 ± 1.9	11.5 ± 1.0	2.5 ± 0.9	3.6 ± 1.0	5.0 ± 1.5	190.6 ± 13.4	> 200

*IC*_*50*_ 50% inhibitory concentration, *CC*_*50*_ 50% cytotoxic concentration, *CC* cardiac cells, *SERT* sertraline, *BZ* benznidazole, *SD* standard deviation, *BT* bloodstream, *MØ* peritoneal macrophage

The drug was also effective against the intracellular amastigotes in peritoneal macrophages, resulting in an IC_50_ value of 1.4 (± 0.6) μM; using intracellular amastigotes in cardiac cell cultures, the IC_50_ value was 6.6 μM (± 1.4). The intracellular amastigotes of the / a Tulahuen strain inside fibroblasts (L929 cells) were also susceptible, resulting in an IC_50_ value of 10.0 μM (± 1.7) (Table 1). The mammalian cytotoxicity was determined in L929 cells and cardiac cell cultures, and resulted in respective CC_50_ values of 11.5 μM (± 2.48) and 25.0 μM (± 13.2). Benznidazole was used as the standard and resulted in IC_50_ values of 17.7 μM (± 1.9) against trypomastigotes (Y strain) and 5 μM (± 1.5) against intracellular amastigotes (Y strain) (Table 1).

### Action mode studies of SERT in *T. cruzi*

#### Permeability of plasma membrane

To evaluate the possible effect of SERT on the plasma membrane permeability of culture trypomastigotes of *T. cruzi*, SERT was incubated for 60 min with trypomastigotes at the corresponding IC_50_ value, after which the permeability of the membrane was evaluated fluorimetrically using the vital dye SYTOX Green®. The data demonstrated no increase in the fluorescence levels, suggesting that sertraline did not interfere with the plasma membrane permeability of the parasite. Triton X-100 was used as a positive control (data not shown).

#### Effects on mitochondrial integrity and ATP production

**Fig. 1:**
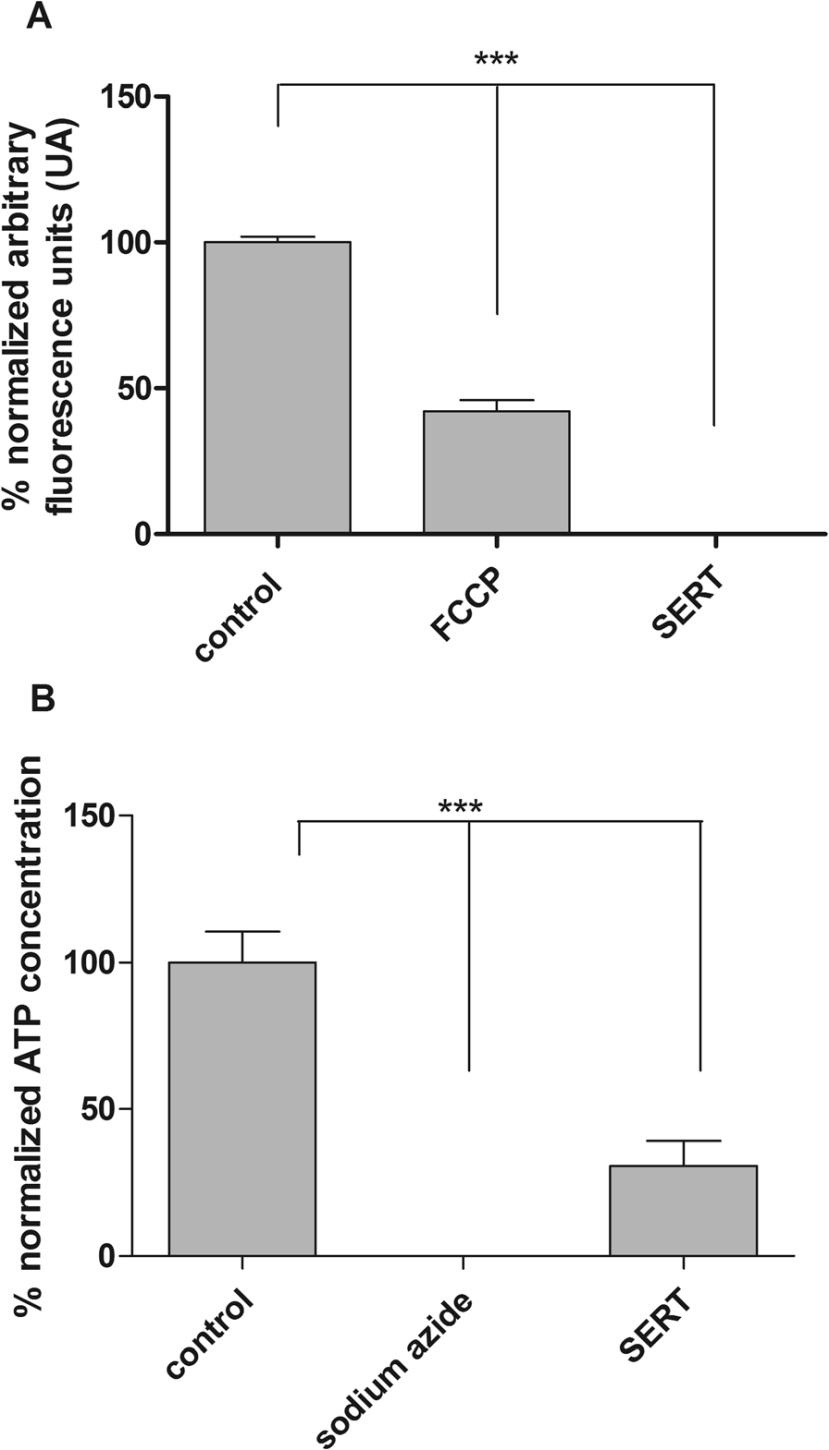
Evaluation of the bioenergetic parameters of *T. cruzi*. **a** Mitochondrial membrane potential of *T. cruzi* trypomastigotes treated with sertraline for 1 h at 2 μM, positive control (FCCP) and negative control (untreated cells). The fluorescence was determined using a fluorimetric microplate reader (FilterMax F5 Multi-Mode Microplate Reader) at 540 and 595 nm for excitation and emission, respectively. *** (*p* < 0.05); **b** Evaluation of the ATP levels of *T. cruzi* incubated for 1 h with sertraline at 2 μM. The levels were measured in a spectroluminometer plate reader (FilterMax F5 Multi-Mode Microplate Reader) using the ATP determination Kit (Life technologies). *** *p* < 0.05

The mitochondrial integrity of trypomastigotes was investigated in the presence of SERT at the IC_50_ value using the fluorescent probe Mitotracker Red®. After 60 min of incubation, SERT induced an intense and significant depolarization (*p* < 0.05), with the fluorescence intensity decreasing by 100% when compared to untreated trypomastigotes (control) (Fig. 1a). FCCP was used as a positive control and reduced the fluorescence levels by approximately 54% compared to untreated parasites. The production of ATP by trypomastigotes was investigated in the presence of SERT using a luminescent assay with luciferase (ATP kit - Thermo). At 60 min of incubation, an intense decrease in ATP levels of approximately 70% was observed compared to untreated parasites (*p* < 0.05) (Fig. 1b). Sodium azide was used as a positive control and resulted in a 100% decrease in the ATP levels of trypomastigotes.

### Reactive oxygen species (ROS) production

Considering the interference of SERT in mitochondrial metabolism, the ROS production by trypomastigotes was evaluated using the fluorescent probe H_2_DCf-DA in the presence of the drug. The results indicated no alterations in ROS levels after 60 or 120 min compared to untreated parasites. Oligomycin was used as a positive control, and it upregulated ROS levels in trypomastigotes, as indicated by increased fluorescence intensity (data not shown).

### In silico studies

To identify SERT targets that were experimentally determined in other organisms, we performed a literature search in PubMed, PubChem Bioassay, BindingDB and ChEMBL. Using a chemogenomic target fishing strategy, we identified 15 similar targets in *T. cruzi* (Additional file 2). Following these conditions, we identified three potential targets of SERT in *T. cruzi* (Table 2).

**Table 2:** List of potential sertraline targets in *T. cruzi*

SERT target	*T. cruzi* target	Biological process	E-value	Overlap	Active Site Conservation (%)
Isocitrate dehydrogenase 1	Isocitrate dehydrogenase 2	isocitrate metabolism	0	100%	High conservation (97%)
Ubiquitin-conjugating enzyme E2 N	Ubiquitin-conjugating enzyme E2, purative	ubiquitin cycle	1^−68^	96%	High conservation (76%)
Cyclin-dependent kinase 1	CDC2-related protein kinase 1, putative	protein phosphorylation	2^− 113^	95%	High conservation (78%)

### Homology modeling and molecular docking

**Fig. 2:**
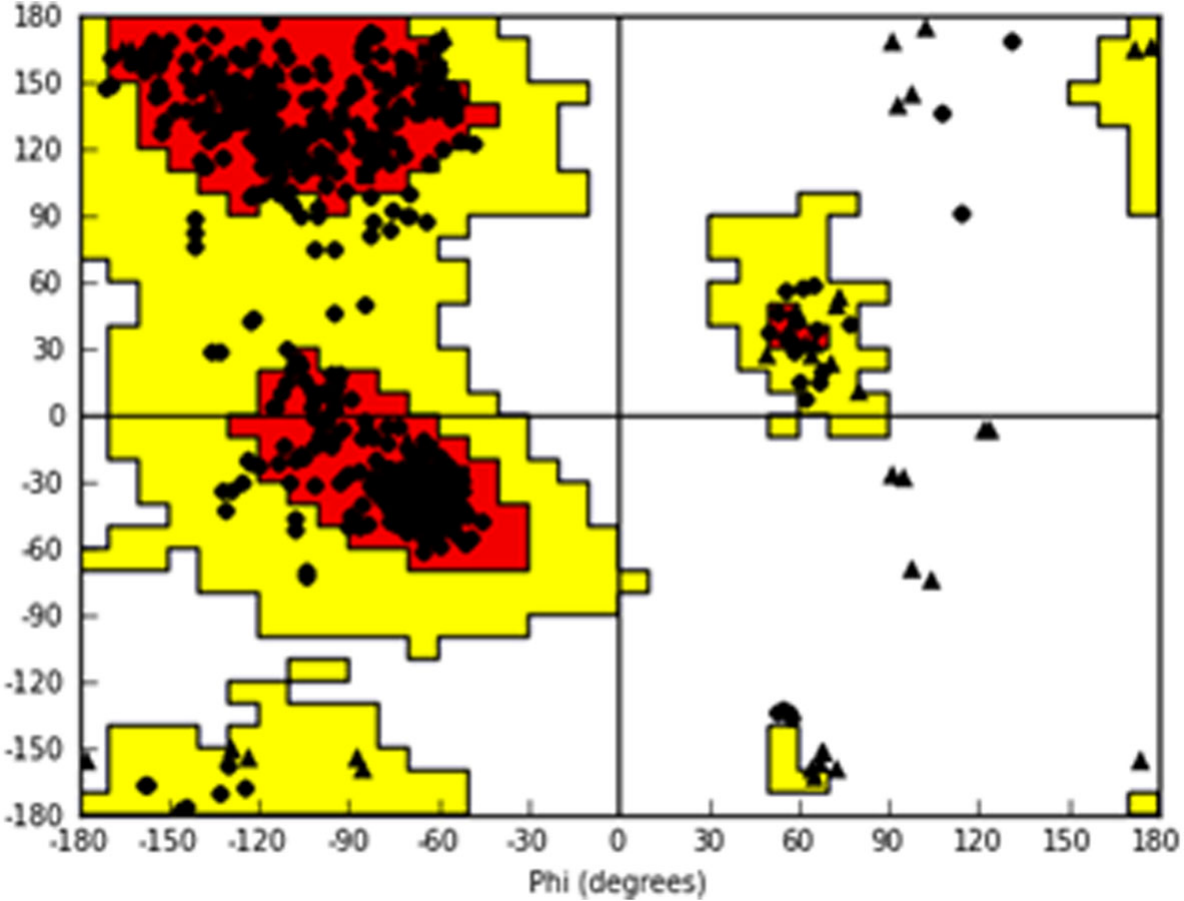
Ramachandran plot for the *Tc*IDH2 homology model. The amino acids are represented as circles, except for glycine, represented as triangles. The white area shows sterically disallowed regions for all amino acids, except glycine. The yellow areas show the sterically allowed regions. The red areas indicate the most sterically favorable regions, i.e. regions where amino acids conformations have no steric clashes

To build the homology model of *Tc*IDH2, the following criteria were used for template selection: the template should have high coverage, good sequence identity and good X-ray crystallography resolution (< 2.0 Å). Therefore, the selected template, *Hs*IDH2 (PDB ID: 4JA8), presented 66.5% sequence identity with *Tc*IDH2, coverage of 0.99 and a resolution of 1.5 Å. The stereochemical quality of the *Tc*IDH2 model was evaluated using PROCHECK. This analysis revealed that 91.7% of residues were within most favored regions, 8.2% of residues were in additional allowed regions, and only 0.1% of residues were in disallowed regions of the Ramachandran plot, demonstrating the good quality of the generated model (Fig. 2).

**Fig. 3:**
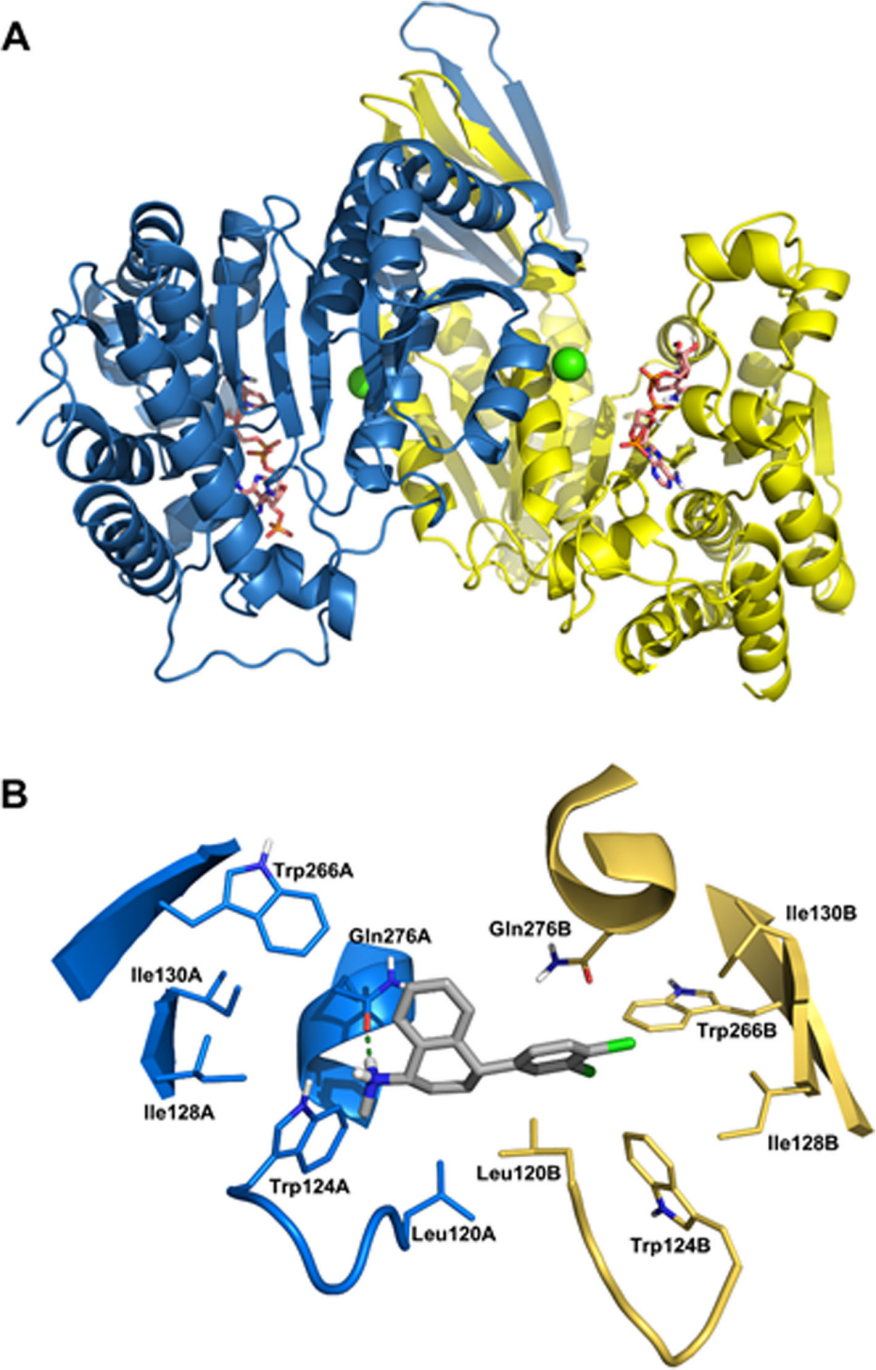
3D structure of *Tc*IDH2 homology model. **a** Regions in blue represent Chain A and regions in yellow represent Chain B. The Ca^2+^ ions are indicated by green spheres. The two ligands presented in the stick model represent the co-factor NADP. **b** Interactions of sertraline with the allosteric binding site of *Tc*IDH2 were predicted by docking. The residues of chain A are in blue and chain B is colored in yellow, indicating the interface that constitutes the allosteric binding site. In the center, sertraline carbon atoms are colored gray, nitrogen in blue, chlorine atoms are green and hydrogens are white. The hydrogen bond between sertraline and Gln276A is indicated by a green dashed line

Additionally, the quality of the homology model was evaluated using VERIFY 3D, through which the *Tc*IDH2 model presented a score of 92.6%. An overall quality factor of 86.6% was obtained from the analysis on ERRAT. The homology model of *Tc*IDH2 had a satisfactory quality and could be used in molecular docking studies. Figure 3a shows the generated 3D structure of the *Tc*IDH2 homology model. Docking studies were performed at two different binding sites of *Tc*IDH2, the catalytic site, i.e., the binding site of the substrate isocitrate, and in the allosteric site, which is located in the interface between the two chains of the homodimeric protein. The predicted binding affinities of SERT in the catalytic and allosteric sites were - 5.5 kcal/mol and - 12.2 kcal/mol, respectively. The remarkable difference in binding affinities indicates that SERT may be an allosteric inhibitor of *Tc*IDH2.

Moreover, docking results showed that the predicted binding mode of SERT at the *Tc*IDH2 allosteric site is in agreement with the experimental X-ray binding mode of the co-crystalized ligand on *Hs*IDH2. In particular, SERT can establish a hydrogen bond between its secondary amine hydrogen and the amino acid residue Gln276A. Additional hydrophobic interactions were observed between the SERT aromatic ring and residues Trp266B, Trp124B and Leu120B (Fig. 3b). The presence of a hydrophobic pocket represented by Leu120A, Leu120B, Trp124B, Ile128B, Ile130B and Trp266B can provide hints for the structural optimization of SERT, thus enabling the design of new inhibitors of *Tc*IDH2.

## Discussion

Drug repurposing is a successful approach with numerous advantages, including reduced time and costs in the drug discovery process. Sertraline, an FDA-approved drug, belongs to the class of antidepressant agents known as selective serotonin-reuptake inhibitors (SSRIs). The literature reports several attempts to repurpose the drug SERT towards a range of microorganisms [32, 33].

Considering the broad range of biological activities of SERT, our work investigated the in vitro anti-*T. cruzi* efficacy against trypomastigotes and intracellular amastigotes. Based on the 50% Inhibitory Concentration (IC_50_), SERT demonstrated similar in vitro efficacy to the standard drug (BZ) against trypomastigotes and intracellular amastigotes (Y strain). The drug effectively eliminated the intracellular amastigotes of different strains and DTUs of *T. crui* [34]. It was active against the Y strain (DTU II) in macrophages and cardiac cells at micromolar concentrations. Sertraline was also effective against intracellular amastigotes of the Tulahuen strain (DTU VI), an important feature for a hit compound. Also, it is important to note that the difference between these DTUs may vary in activity and therefore should be taken into account in the testing of new drugs [35]. The Y strain was approximately 7-fold more susceptible to SERT than the Tulahuen strain. In addition to its intracellular activity against amastigotes, SERT has been valued as a hit candidate for presenting activity against the trypomastigote forms. Katsuno and co-workers [36] stated that drugs that only target the replicating stages of the parasite may leave non-replicating forms, such as trypomastigotes, capable of maintaining infections long after the end of the treatment; i.e., they are a clinical form responsible for relapses.

One of the advantages of repositioning FDA-approved drugs is the existing medical data on the drug, including dosing, side-effects, tolerance profiles and the pharmacokinetic and pharmacodynamics parameters (PKPD). Although sertraline demonstrated in vitro activity and selectivity against *T. cruzi* in our study, one should consider that for *in vivo* efficacy, adequate tissue distribution and serum levels must be achieved to eliminate the parasite. In the literature, SERT administered in humans resulted in serum levels of approximately 58 nM [37], a considerably smaller value than those needed to eliminate *T. cruzi* intracellular amastigotes (or bloodstream trypomastigotes) in our in vitro assays. Future experimental association studies should be performed to assess sertraline and benznidazole; if synergic combinations are found, reduced doses may be required to treat *T. cruzi* infected animals. Our data also suggest that sertraline may be a useful candidate for both acute and chronic phases of the disease, as the drug is effective against bloodstream trypomastigotes and intracellular amastigotes. However, considering that sertraline is metabolized to desmethylsertraline in the liver, the *in vivo* efficacy of the drug may be evaluated in the future using both mouse models (acute and chronic).

Considering the promising in vitro efficacy of SERT in *T. cruzi* parasites, we investigated the possible mode of action using different approaches, including cell biology techniques and in silico approaches. Using short-time incubations to evaluate initial cellular damage in *T. cruzi*, SERT induced a change in the mitochondrial integrity of trypomastigotes, resulting in a significant decrease in ATP levels within 1 h without affecting plasma membrane permeability. The depletion of ATP is an early event in mitochondrial dysfunction, which generates irreversible damage and cell death [38].

Trypanosomatids display a single mitochondrion with several peculiar features, such as the presence of different energetic and antioxidant enzymes and a specific arrangement of mitochondrial DNA (kinetoplast DNA) [39]. Due to mitochondrial differences between mammals and trypanosomatids, this organelle is a favorable candidate for drug intervention. Multiple mechanisms and targets are often involved in drug- or xenobiotic-associated mitochondrial impairment [40].

In protozoan parasites such as *L. donovani*, the action mode of sertraline was attributed to the decreased cytoplasmic ATP levels and oxygen consumption rate in promastigotes, suggesting an apoptosis-like death in treated parasites [11]. Previous studies have suggested that the decrease in oxygen consumption and reduction in cytoplasmic ATP levels, resulting from the inhibition of the respiratory chain, are essential events at the beginning of apoptosis in *L. donovani* [41–44]. The effect of sertraline in mitochondria was also demonstrated in hepatocytes. According to Li and co-workers [45], SERT began to decrease ATP levels in as early as 30 min in rat primary hepatocytes, targeting complexes I and V in the mitochondria. These data also corroborate a previous study of SERT that resulted in concentration-dependent mitochondrial swelling in hepatocytes [45]. In our study, SERT also seems to affect the mitochondria of *T. cruzi*. Whereas SERT disturbs the bioenergetic system of eukaryotic cells, including *Leishmania*, the standard drug benznidazole demonstrates a different action mode in *T. cruzi*. It is important to note that the action mode of BZ is apparently related to the formation of free radicals and electrophilic metabolites that are generated when its nitro-group is reduced to an amino group by the action of nitroreductases [46, 47]. Thus, it is hypothesized that the trypanocidal effect of BZ is caused by the covalent attachment of its reduced metabolites to macromolecules of the parasite [48].

Currently, several in silico chemogenomic strategies have been applied to drug repositioning against parasitic diseases. The main goal of the chemogenomic approach is to identify new therapeutic targets and drugs. To achieve that goal, various public drug databases that integrate information about gene/protein–drug–disease interactions, such as the Therapeutic Target Database (TTD), DrugBank and STITCH, are valuable resources to develop these strategies. Based on the concept that “similar targets have similar ligands”, homology-based inverse virtual screening allowed the prediction of potential targets of SERT in *T. cruzi* [49].

To identify SERT targets that were experimentally determined in other organisms, we performed a target fishing approach using a literature search in PubMed, PubChem Bioassay, BindingDB and ChEMBL. Using this chemogenomic strategy, we identified 15 similar targets in *T. cruzi* (Additional file 2). Most target databases are only starting to emerge, and the predicted *T. cruzi* targets are not yet scored for druggability. The druggability concept adds a structural dimension and evaluates the likelihood that small drug-like molecules can bind a given target with sufficient potency to alter its activity [50]. Therefore, the predicted *T. cruzi* targets were considered druggable if they presented an overlap ≥80% of the SERT target, an E-value ≤10^− 20^ and conservation of the functional regions. Thus, overlap sequences and conserved functional regions analysis of positions among SERT targets and *T. cruzi* targets revealed the importance of each position for protein function and also the possible preservation of affinity for SERT.

Among the predicted targets, isocitrate dehydrogenase 2 (IDH2; Accession: Tc00.1047053506925.319), an enzyme that catalyzes the oxidative decarboxylation of isocitrate to produce 2-oxoglutarate, CO_2_ and NADPH [51], was suggested. A search of the *T. cruzi* genome database (http://www.genedb.org/Homepage) showed the presence of two IDHs, namely IDH1 and IDH2. Both IDHs exhibited remarkable relatedness (> 65% identity) and revealed an equivalent degree of similarity compared to the NADP-linked mammalian counterparts, such as human IDH1. In *T. cruzi*, IDH1 is restricted to the tricarboxylic acid cycle (Krebs cycle) and provides a portion of the NADH utilized for ATP production by oxidative phosphorylation [52]. In contrast, NADP-linked IDH2 localizes in the peroxisomes, mitochondria and cytosol, where their biological roles are associated with multiple functions, such as intracellular redox homeostasis, β-oxidation of fatty acids, and lipogenesis. Furthermore, the expression levels of IDH2 are significantly increased in amastigotes and trypomastigotes compared to epimastigotes. Conversely, IDH1 appears to be more abundant in the insect stage of *T. cruzi* [51]. Therefore, our in silico study suggests that SERT is able to inhibit *T. cruzi* IDH2, a homologue of human IDH1, with higher overlap and conservation of the active site than the other predicted targets.

In addition to the targets highlighted above, 15 other *T. cruzi* targets were similar to the SERT targets in other organisms. In all cases, we considered the numerical parameters (overlap, conserved functional regions, and E-value) for target homology to be sufficiently significant to infer the predicted target with a high degree of confidence. They were not discussed in detail due to their low druggability profile. For example, *T. cruzi* mitogen-activated protein kinase (E-value = 5^− 91^; overlap = 95%) has low conservation of functional regions (61% of predicted residues are conserved), which is insufficient to infer the predicted target with a satisfactory degree of confidence.

## Conclusions

Our studies demonstrated that SERT has a rapid and lethal effect on different forms and strains of *T. cruzi*, affecting the bioenergetic metabolism of the parasite. The depletion of ATP levels in trypomastigotes and the change in mitochondrial integrity may be initial effects of the drug, contributing to the death of *T. cruzi*. However, considering the multi-target characteristic of SERT, our in silico studies also suggested that sertraline affects the parasite *Tc*IDH2, a parasitic enzyme possibly involved in the mechanisms of resistance to oxidative stress. Due to the high homology between the *Tc*IDH1 and *Tc*IDH2, SERT may affect both enzymes. Therefore, further in vitro enzymatic studies involving the predicted enzymes are required to confirm the potential inhibitory effect of SERT. Considering our in vitro experimental studies and the homology-based inverse virtual approach, sertraline should be considered a new hit compound for *T. cruzi*. These findings provide a starting point for future experimental assays and may contribute to the development of new compounds.

## Additional files

Additional file 1: Flowchart of the global methodology (PNG 147 kb)

Additional file 2: List of compiled and predicted *T. cruzi* targets (XLSX 166 kb)

Additional file 3: Alignment and functional region analysis of the *T. cruzi* IDH2, purative (Sbjct) and human IDH1 (Query) (PNG 312 kb)

Additional file 4: Alignment and functional region analysis of the *T. cruzi* ubiquitin-conjugating enzyme E2, putative (Sbjct) and human ubiquitin-conjugating enzyme E2 N (Query) (PNG 143 kb)

Additional file 5: Alignment and functional region analysis of the *T. cruzi* CRK1 (Sbjct) and human cyclin-dependent kinase 1(Query) (PNG 238 kb)

Additional file 6: Alignment and functional region analysis of the *T. cruzi* mitogen-activated protein kinase (Sbjct) and human mitogen-activated protein kinase 1 (Query) (PNG 277 kb)

## Abbreviations

ATP: Adenosine triphosphate
BT: Bloodstream trypomastigotes
BZ: Benznidazole
CC: Cardiac cell
CC_50_: 50% cytotoxic concentration
DMSO: Dimethyl sulfoxide
DNDi: Drugs for Neglected Diseases Initiative
FBS: Fetal bovine serum
FCCP: Carbonyl cyanide 4-(trifluoromethoxy)phenylhydrazone
FIOCRUZ: Fundação Oswaldo Cruz
HBSS: Hanks’ Balanced Salt Solution
IC_50_: 50% inhibitory concentration
NTDs: Neglected Tropical Diseases
PBS: Phosphate-buffered saline
ROS: Reactive oxygen species
RPMI: Roswell Park Memorial Institute Medium
SERT: Sertraline
TTD: Therapeutic Target Database
