# Supplementary figures and images for: Efficacy of sertraline against Trypanosoma cruzi: an in vitro and in silico study
Source: J Venom Anim Toxins Incl Trop Dis. 2018 Oct 30;24:30. doi: 10.1186/s40409-018-0165-8 (PMC6208092; doi:10.1186/s40409-018-0165-8)

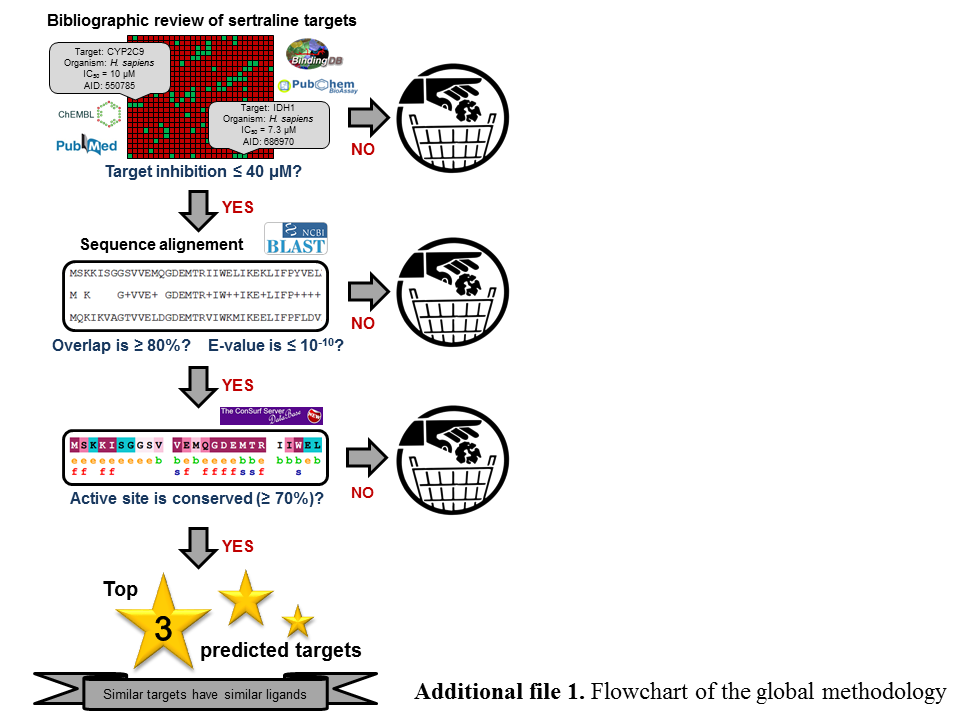

Supplement: Supplementary file 1 — Flowchart of the global methodology (PNG 147 kb) [file 40409_2018_165_MOESM1_ESM.png]

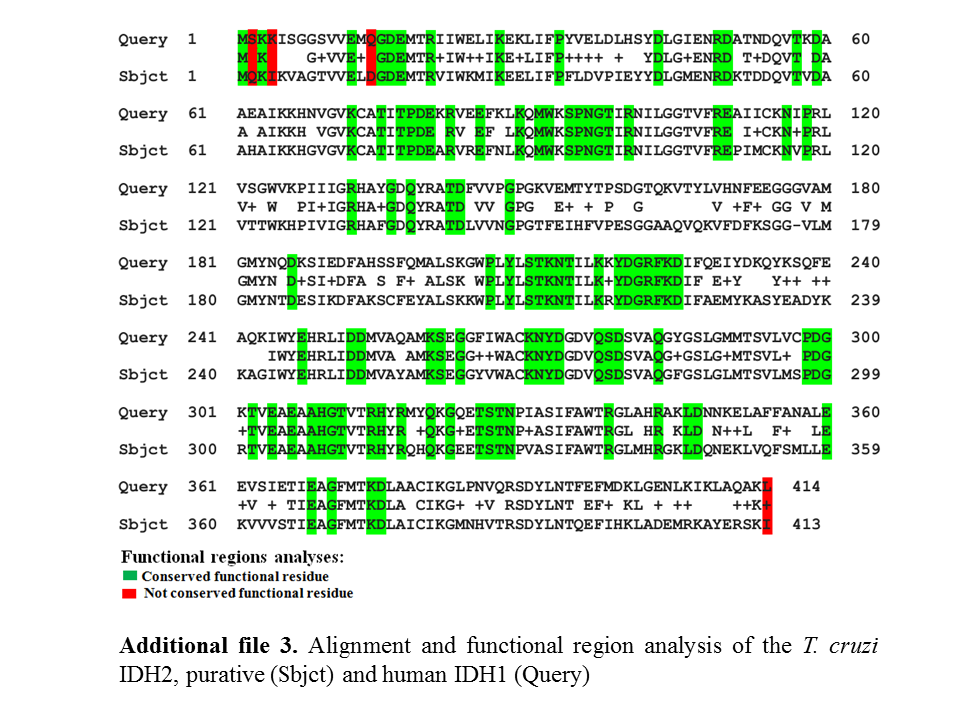

Supplement: Supplementary file 3 — Alignment and functional region analysis of the T. cruzi IDH2, purative (Sbjct) and human IDH1 (Query) (PNG 312 kb) [file 40409_2018_165_MOESM3_ESM.png]

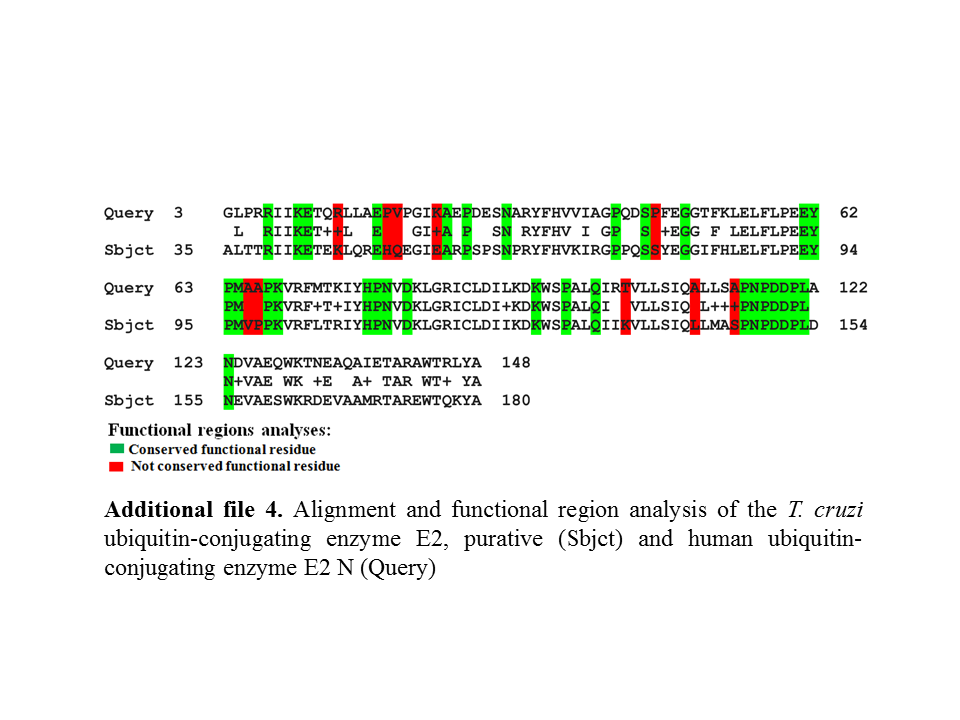

Supplement: Supplementary file 4 — Alignment and functional region analysis of the T. cruzi ubiquitin-conjugating enzyme E2, putative (Sbjct) and human ubiquitin-conjugating enzyme E2 N (Query) (PNG 143 kb) [file 40409_2018_165_MOESM4_ESM.png]

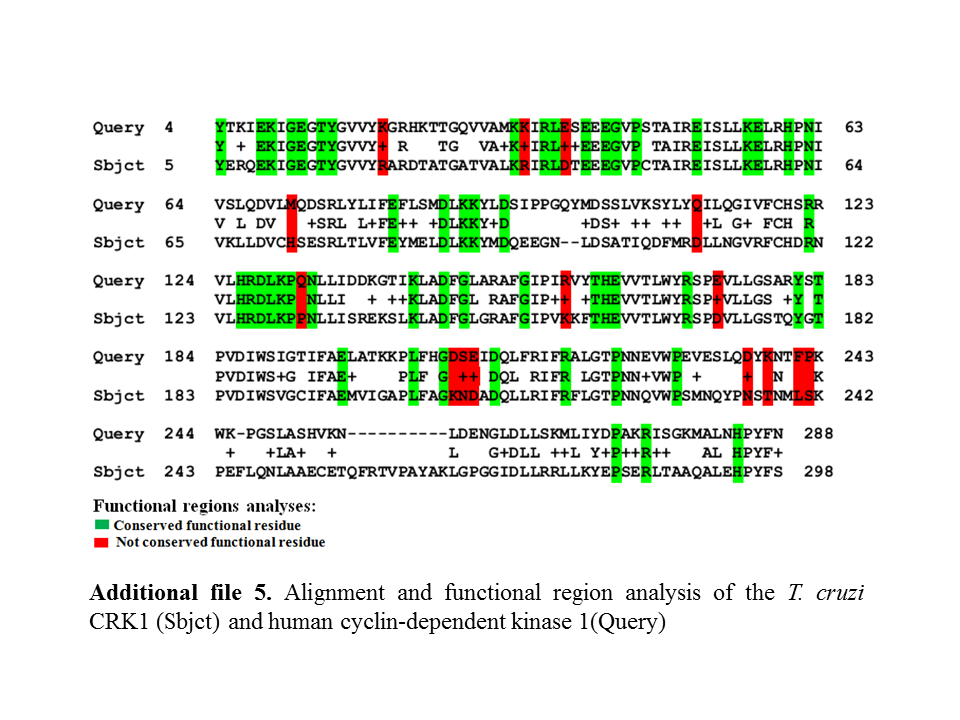

Supplement: Supplementary file 5 — Alignment and functional region analysis of the T. cruzi CRK1 (Sbjct) and human cyclin-dependent kinase 1(Query) (PNG 238 kb) [file 40409_2018_165_MOESM5_ESM.png]

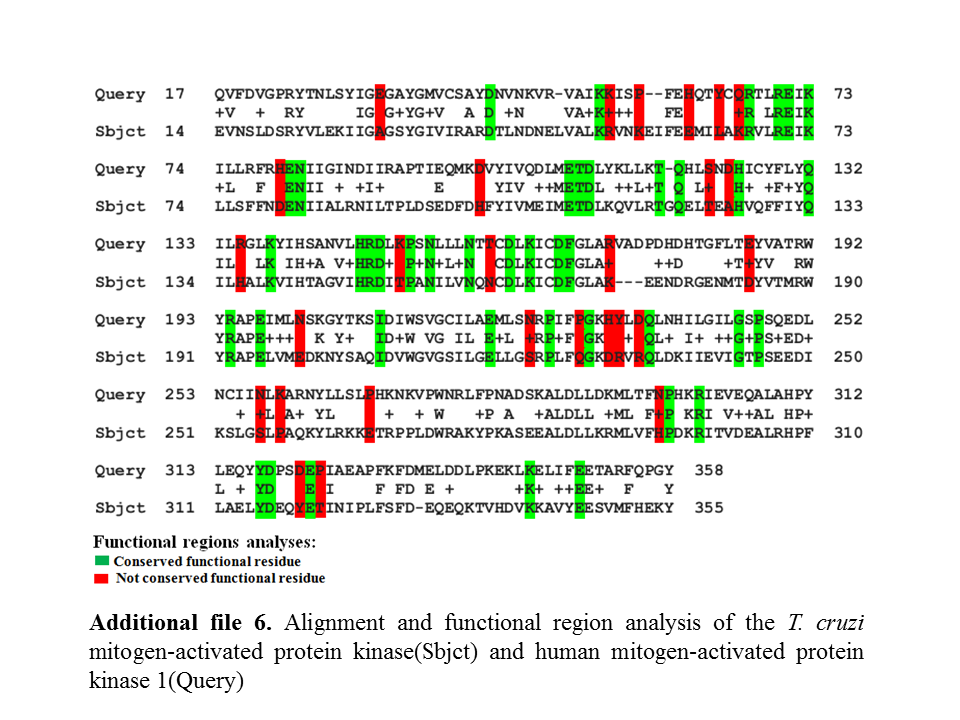

Supplement: Supplementary file 6 — Alignment and functional region analysis of the T. cruzi mitogen-activated protein kinase (Sbjct) and human mitogen-activated protein kinase 1 (Query) (PNG 277 kb) [file 40409_2018_165_MOESM6_ESM.png]
